# Miscellanies

**Published:** 1834-04-01

**Authors:** 


### Miscellanies.

### Foreign Medical Politics.

The following extracts from the report of a committee of the Academy of Medicine in Paris, upon a plan for the reorganization of medicine in France, will be read with great interest at the present period. They have been furnished us by Dr. Thomson, M.B. of St. John's College, Cambridge, now residing in Paris; and are also published in our contemporary, the London Medical and Surgical Journal—

"The question put by the government is thus expressed:—

'Can we, without inconvenience, renounce having two orders of medical men?'

To answer it deliberately, let us see first what existed formerly in France, compared with what exists in the present day, and contrasted with the medical institutions of neighbouring nations.

Preliminary studies, long and solid, a diploma, required in the Faculty of Letters, and recently even another diploma, required in the Faculty of Sciences; four years' inscriptions taken in a Faculty; five examinations, crowned by an inaugural thesis; expenses, which, for the University rights and the diploma, amount to 1,100 francs (about fifty pounds sterling). Such are the obligations that must actually be fulfilled by the Doctors in Medicine, or in Surgery.

Preliminary studies, none or insignificant, three years' study in a Faculty, or in a secondary school, which may be replaced by six years' presence in an hospital, or studies with a doctor; three examinations, most frequently illusory; an outlay amounting, for the University rights and the diploma, to 250, or at most 300 francs (about ten or twelve pounds sterling), there is what is required from the officiers de santé (a species of general practitioners.)

To the first is reserved the right of practising to the fullest extent throughout the kingdom. The rights of the second have been in some respects limited, but the restrictions have been constantly illusory: the Doctors of Medicine recoiling before the anxiety and scandal of enforcing their rights by law-suits, and even the tribunals hesitating to apply the law in all its rigour."

"Let us now consider what takes place in neighbouring nations. In England the apothecaries have the right of practising and of prescribing the remedies they prepare; they, in truth, form a class of practitioners inferior to the physicians.

In Prussia, Germany, and Italy, the physicians and surgeons are admitted separately; but every where, as formerly with us, with the exception of some of the heads elevated to the first rank by their talent, the surgeons in general, and even some of the medical men, compose the inferior class, and serve most commonly as assistants and servitors to the others.

Thus, in this general review, we meet every where with two orders of practitioners. Must we conclude that such a state of things ought to be our guide, and that the past ought to form in this case a law for the future? Quite the contrary: since this past no longer corresponds to the irresistible wants of a new state of society; since, on all hands, there arises an unanimous outcry, it is too evident that we must seek in this organization itself, for the cause of this restlessness which torments us; and that the errors of the past ought to serve us as lessons. Called upon especially to correct the defects of the ancient legislation, the new legislation must, before every thing, prove its superiority to the other; it is only by deviating from the route hitherto followed that we can act otherwise and better.

And first, this idea of creating by a law two orders of medical men unequal in rights, in instruction, in capacities, is manifestly repugnant to reason and to justice. Humanity even is seriously injured by it. What! shall there be one part of the population to whom shall be reserved all the resources of the art of healing, and another part abandoned, *à priori,* to the errors, the faults, the ignorance of a class of inferior practitioners? Such a distinction is not admissible in France, —it would be odious—it is absurd. In place of seeking to diminish intelligence by imposing on it an inferior level, we must try to elevate it more and more; the science must be accessible to all, but all must be obliged to cultivate equally the science. Our faculties, with the conditions they require from their pupils, with their immense means of instruction, with their numerous examinations, can scarcely ever arrive at producing medical men, not inferior to their mission. How could it be wished to entrust the health of citizens to officiers de santé, deprived at once of the preliminary knowledge indispensable to medicine, and of the means of study; and to hasten to receive them without giving them the time to study. If in all arts half knowledge is injurious, *à fortiori* in medicine, in which the least errors may become irreparable and endanger the life of citizens.

To create superiority by an article of law is absurd, and repugnant to the nature of things. The defenders of the institution of the officiers de santé say, that there is no need of such high medical qualifications for the country; let them rest assured mediocrity will never be wanting. It is a law of humanity which the legislator cannot remedy; but it is his duty to stipulate for society all the guarantees that are at once possible and necessary. All these guarantees are contained in the diploma of doctor, which doubtless cannot give to all an equal capacity, but which gives to all the same legal value; as the stamp to gold, as the effigy of the prince to the coin of the kingdom.

Such are the reasons in favour of the suppression of the officiers de santé. But objections against this measure have not been wanting; we must now appreciate their value. First, they say to us, if you exact for a diploma of a medical man expenses so considerable, both of time and money, it will happen that many strong profound intellects will be forced from this career. Thence a double disadvantage, on the one hand, for individuals whose prospects will be lost; on the other, for the science, on which talents you reject might have impressed an increasing and glorious progress.

Further, these expenses, to which you will subject small fortunes, will necessarily induce the very natural desire of a proportionate remuneration; and as great towns only have the privilege of offering a brilliant prospect of ambition, this mass of doctors you are about to create, will crowd into the great towns, and leave the country places abandoned to quacks, who have not even intentions to allege in their favour, or else to sisters of charity, of whom the very praiseworthy zeal cannot disguise their ignorance and incapacity.

The objection reduces itself to this, that the too elevated prize of the doctorship will repel many men who might have been the glory of the science, and abandon country places to quacks.

The answer is easily given. And first, to arrive at a profession, promising at once ease, glory, and as much independence as is desirable from any other, without running risk to property or honour, assuring to all its members a good position in society, and, finally, an existence at least tolerable, is there really too great a sacrifice in four or five years of study and eleven hundred francs of expense? At a period when a numerous youth encumbers every career, when fortunes, rendered equal by the division of lands, by giving to all fathers of families the power of making economies, have inspired the taste, are we justified in fearing that medicine will want aspirants, and that the so moderate exigencies of the law obstruct too closely its entrance? Where is the profession placed as high in the social scale, which has need of fewer aspirants? We find every where the time of probation, under different names, the supernumerary period, the clerkship, &c."

"We impose too great expenses! In truth this is laughable. But in the other professions, the outlays, the caution money, the purchases of practices of lawyers, and of merchandize, are there not advances far more considerable? And then, moreover, it is easy still to reduce these expenses; multiply the places of instruction; let the young people find nearer their families the instruction they are obliged to seek at such great distances.

For our part, what we alone wish, what is important to us, are guarantees; and on that account we call for more rigorous and more difficult examinations than at present exist, and do not fear, in proportion as the trials shall be more severe, that the candidates will be disgusted, and their number diminish; *the rigour of the examinations of the Polytechnic school, by augmenting the consideration, reflected upon the candidates admitted, has only increased the emulation and the number of aspirants.*

But, further, to remove such an objection, are our Faculties more deserted? Never was the influx so considerable. Will a complaint perchance be made of a want of medical men? With more justice is their too great number complained of. It is moreover said, that the rural parishes *(communes)* possess, generally, too little wealth, and instruction, and even distinction, to satisfy the intelligence and emulation of a doctor of medicine. What then! will it be said that the *officiers de santé* are less sensible than the doctors to all these advantages? The proof of the contrary is every day before our eyes; officiers de santé have quitted country places for towns; they practise in them on an equality with the doctors; they take great care to have themselves equally remunerated.

One of the wants of medicine in the present day is, beyond contradiction, a more equal distribution of medical men relative to the population, and, at the same time, with a more equal distribution of instruction among the profession. Medicine is not only an art, it is also an occupation, that should yield for each service rendered its reward. Doubtless it would be desirable to arrive at this end, to enrich the poor, and people the desert parts of the country; but if these ameliorations can only be effected after a long lapse of time, there are yet certain prudent measures which may, to a certain extent, supply their place. Thus the number of doctors will be on the increase in country places when they shall no longer fear being confounded with the *officiers de santé;* when each of them, penetrated with the dignity of his profession, shall no longer see arising near him an ignoble rivalry, and the science offered at a lower price. It must be confessed, indeed, that the greater part of the *officiers de santé* not having for their guide the recollection of a good education, do not always follow, in their private conduct, the most honourable course; and as to their science, far from augmenting the little knowledge they may have acquired, the isolation in which they live makes them frequently forget it too speedily. Hence a justly-founded repugnance on the part of the doctors to mingle with such men. Put an end to this cause of their absence, and be sure that the country places will not remain long without medical men."

It may be well worth while to compare and contrast these liberal sentiments with the tortuous, constrained, and absurdly multifarious system of medical polity adopted in that model of free nations—Prussia!—a system, the *principle* of which is recommended for adoption in this country by our contemporary, the Medical Gazette. Let Englishmen read and judge.

"The medical profession in the Prussian states is divided into the following orders:—

I. *Graduate Practitioners of Medicine.*

This, the highest class, comprehends two subdivisions;—

1. Those who are graduates in surgery as well as in physic, and who thus unite in themselves whatever the medical art embraces in its entire extent. They are physicians and surgeons (*chirurgico medici, chirurgiatri*), or physicians and operators together. And in order to attain this rank, they are required,—

(*a.*) To have regularly taken their degrees as doctors of medicine and surgery. The term *regularly* here implies that the person shall have obtained the doctorate, after having in the first instance given proofs of a sufficient school education; then by going through a four years' curriculum in a University, commencing it with a *Tentamen philosophicum,* passing an examination before the faculty, and in conclusion, defending publicly an inaugural dissertation composed in Latin by himself. Then he shall shew that he has gone through with success, (*b.*) The required course of anatomy; (*c.*) Akiurgy, (mechanical or operative surgery); (*d.*) Clinical surgery; (*e.*) An examination on clinical medicine, conducted in the Latin language; and (*f.*) The oral final examination, that for the diploma; the extent of this trial takes in the whole range of the healing art.

Having complied with all these requisites, the candidate takes the oaths, and receives his diploma; and he thus becomes entitled to turn his acquirements to account, by exercising the medical art in all its branches. The minor surgery alone and minor offices, graduates of this high order, are obliged to relinquish for the benefit of the pure surgeons in those places where such are settled; those cases of course being excepted, where the occurrence of delay would be dangerous.

Whether the practitioner belonging to this class shall or shall not have the the additional title of operator, depends chiefly on the result of his akiurgical and clinico-chirurgical examination.

2. Those who are graduate practitioners of medicine only (*medici.*) These are confined to the practice of inner medicine (*innere Heilkunde*); but they are by no means allowed upon this account to dispense with a knowledge of surgery: they are only not permitted to practise it, nor are they required to give proof of their practical ability in this respect.

To obtain the diploma of a practising physician of this class, the candidate must shew, (*a.*) That he has regularly graduated as a doctor of medicine; (*b.*) That he has gone through with success the anatomical course; (*c.*) The clinical-surgery examination; however, with reference only to the pathological part of surgical diseases, omitting the operative detail; (*d.*) The clinical-medicine examination in Latin; and (*e.*) The oral concluding examination, in which the candidate shall be examined, among the rest, on the nature and treatment of surgical diseases.

The persons who belong to this order of the profession, comprehended under the two preceding subdivisions, besides the privileges already mentioned, are alone qualified for appointments as medical officers of state, from a chief medical privy councillor down to a physicus, provided they have previously given proof of the requisite knowledge in midwifery, have performed their medico-legal exercises with approbation, and have gone through the stated examination for the physicate. It is also only graduate practitioners of medicine who are eligible to the higher medical professorships; and in the military medical service, it is only those who belong to the first subdivision who may obtain the higher professional appointments, from staff-physician-general down to regimental physician.

II. *Surgeons of the First Class.*

These are medico-chirurgi, or iatrochirurgi, who have not taken a degree. Individuals of this order must possess the requisite knowledge for treating inner as well as outer maladies, according to the precepts of the schools. They must, therefore, in order to be admitted to an examination, and to be qualified for their diploma, shew (*a*) by gymnasial certificates, or a preliminary tentamen, that they possess the requisite general elementary knowledge, and at least so much Latin as to be able to translate the Pharmacopœia and some easy author, and to write a prescription correctly. Further (*b.*), they must shew either that they have gone through a regular three years' medico-chirurgical course, and have obtained the requisite practical ability from clinical instruction, or that they have attended the prescribed courses of lectures, and have for the same length of time, at least, acted as assistant-surgeons in a military or civil capacity. Then they must have gone through, with success, (*c.*) the anatomical course, (*d.*) akirurgy, (*e.*) clinical surgery, (*f.*) the medico-clinical examination in German, chiefly touching acute diseases, and of a purely practical tendency; and (*g.*) the oral final examination for the diploma, which shall be as well on medical as on surgical and pharmaceutical subjects.

Whether they shall obtain the additional title of operator, depends on the issue of their examination in practical and clinical surgery. But the privilege of medical and surgical practice accruing to this class of surgeons, depends on the following external circumstances. It is only when they choose to settle in a place where there is not already a graduate practising physician, that they may practise medically; but the privilege continues with them, although a graduate may subsequently come to settle in the same locality. If, however, they wish to practise in large towns, or where there are already graduates residing, and there be no need of a medical assistant, they are then only allowed to treat surgical cases, and purely medical practice is forbidden them. An exception is made in favour of those surgeons of the first class who hold military or civil appointments—such as battalion physicians or district surgeons; to whom, in consideration of their not being at liberty, in the first instance, to choose their own locality, it is conceded to practise medically as well as surgically, in all places wherever they may be, and as long as they are in the service of the state. For the rest, the surgeons of the first class are bound, in cases of consultation, to pay due respect to the opinions and suggestions of the graduate doctors.

Surgeons of this class being appointed chiefly with reference to their practical utility, with a view to the supplying, by their means, the country folk and inhabitants of small towns with proper medical assistance, are alone held eligible to appointments as district surgeons, when they shall have previously obtained a midwifery license, and passed successfully the medico-legal examination;—as also they may be promoted to the office of surgical assessors in the medical colleges, to district pauper surgeoncies, to be assistant physicians and surgeons in hospitals, and, in the military service, battalion, garrison, and government staff surgeons.

An objection has been made to the introduction of this class of the profession (which has, in point of fact, proved itself to be the most truly useful, perhaps, of all), that, 1, there is thus provided for the people in the country a less competent class of medical practitioners than for those in town; 2. that when these surgeons are held competent for internal practice, it is unfair to refuse them the liberty of practising medically in large towns; and, 3, that the well-earned privileges of the graduate doctors are prejudiced and injured. All this, however, is founded in mistake. The surgeons of the first class, though only allowed the title of surgeons, are just as competent for universal practice as the graduate doctors. It is not the proof of their acquirements adapting them for universal practice that is remitted to them, but that learned education requisite for the cultivation of science: besides, the examination required of both is exactly the same. It is not to be denied that a man may be a very useful and most successful practising physician, without having any pretensions, at the same time, to a learned education, or being competent to promote medical science. On the other hand our most learned physicians, the heroes of science, are often not the best practitioners: one may thus be both a learned and a practising physician, but also either separately. So far, then, as mere ability for inner practice is concerned, there would be no reasonable ground for refusing to surgeons of the first class the right of treating medically the dwellers in large towns—a right which is enjoyed by surgeons of the same class when in official situations.

But the object was to provide the country folk and inhabitants of small towns, who were so frequently destitute of genuine medical assistance, with a better instructed and more variously accomplished class of practitioners than they had under the old system; and for great towns, where professional labour can be liberally remunerated, to secure the required supply of authorized assistant-surgeons, and thus again to put a stop to the bunglings of the mere barbers. For this reason, it was necessary to lay down certain restrictions for surgeons of the first class, with regard to their practising medically; such as that they were to be recognized as medical practitioners only when there was a lack of that class—because they would otherwise (like the licentiates, their predecessors) take good care not to settle in the country, where they would have less profitable and far more arduous duties imposed upon them than in town.

The objection, then, that it is unjust to forbid this class of medical practice in large towns, at once disappears. It is only to the surgeon of the first class who is in the service of the state, that this privilege can be permitted; because, otherwise, such an individual suffering a greater loss by the prohibition of the privilege of inner practice than the gain that accrues from his very parsimonious pay, would gladly remain unattached to the service, and the state would soon find itself constrained to fill up its appointments with an inferiorly qualified class of surgeons; and that, too, in the large towns themselves. And, in reality, the country, as well as the military service, requires a class of professional men specially qualified for the duties. The graduate doctor, if he be not at the same time a surgeon, can be as little serviceable in military service as in the country, and, in general, cannot get a livelihood in the latter without some official appointment at his back. Neither his taste for intellectual society, nor his endeavour to keep pace with the advancement of science, nor the pretensions which he can reasonably make to a better way of living can here find their contentment. By the multiplying of graduate doctors, the towns would be overstocked with them, whilst the country would remain unprovided with the requisite medical aid; as the experience of various inland parts can testify. Now this defect could only be remedied by the introduction of a suitable class of practitioners, who, by reason of an inferior scientific education, a less expensive mode of attaining their professional standing, and by reason also of the lower condition of life from which, for the most part, they are sprung, have thus fewer demands and less pretensions to affect a station and rank in society. For the rest, it remains undeniable, in spite of the many objections urged to the contrary, that the country physician, like the military physician of lower rank, requires no such great extent of scientific attainments for the sort of inner medicine which falls to his share: not so extensive, at least, as the practitioner in town. The whole host of chronic and acute disorders which are introduced by a sedentary life—by the refinements of cookery—by the ever-varying fashions in dress—and by the luxurious habits in towns—are only of rare occurrence in the army, as well as in the country. A more uniform way of life, similarity of occupation, and in part an equality of age, give room for a more uniform appearance in the complaints, and preclude those endless complications which are owing to the influences just alluded to. And in addition to this, the physician in a great city must have acquired, besides his professional knowledge and experience, an abundance of general knowledge and maxims of life, if he wishes to move in the higher circles of society, and to inspire confidence in his skill. All which are no ways essential for the country practitioner.

In fine, as to the objection that by introduction of surgeons of the first class, the well-earned rights of the graduates are prejudiced, the repetition of what has been said must be a sufficient reply—that this is the case in a much less degree than it formerly was, when ungraduated physicians (the licentiates) were permitted to practise; for they possessed, in every respect the same rights and privileges as the graduates did. Moreover, the interests of the profession were much less to be considered in the arrangement than those of the sick, who wanted medical aid and had none. Yet with all this, the doctors, so far from having actually lost by the introduction of surgeons of the first class, have in reality gained by it; for there has resulted from that step a more uniform distribution of medical practitioners, and a limit has been set to their superabundant influx in large towns. By the simultaneously introduced difficulty of accomplishing the University course—by the perfecting and extending of the higher medical curriculum itself—and by the severity of the subsequent examinations for attaining the doctorate—the number of graduates has been very much diminished. Each person who, at present, can only reach the rank of surgeon of the first class, could, on the old system, have graduated, as competent for inner practice; or he went through the examination for the licentiateship, and then took his degree into the bargain, without, after all, possessing more acquaintance with the science of his profession than does the present surgeon of the first class. Only the name and the privilege have been altered, not the thing itself. Thus, by the introduction of surgeons of the first class in the Prussian States, there has not been one more practising physician added than there would have been without this class; and only the number of *unlearned* doctors has diminished in proportion as there has been a vent opened for them among the first class surgeons.

III. *Surgeons of the Second Class.*

The candidate who wishes to obtain a diploma as surgeon of the second class, must shew either that he went through the education and apprenticeship prescribed by the medical edict of 1725, or that he had been a military assistant-surgeon at least for three years, or that he had obtained the knowledge and expertness requisite for a surgeon of the lower rank, by the regular attendance of public places of instruction. In the latter respect the candidate must prove that he has gone through with success a complete (three years) curriculum in a native medico-chirurgical establishment (School of Surgery). Yet will certificates of other lectures, attended even in foreign establishments, be admitted as valid, among which, however, those on bandages and the use of instruments, on fractures, and luxations, on operations, and surgical clinic, cannot be dispensed with; and together with which it must be also made to appear, that the candidate attended the said clinic not merely as a hearer, but as a practical assistant;—that he performed dissections, and took part in the practice of operations on the dead body, and on models *(Phantome.)* With these testimonials, the candidate will be admitted by the Medical College of his province to an examination, which is to be completed in four terms.*"

* Medical Gazette, No. 323.

From such a farrago of divisions and subdivisions in British medicine, "good God *deliver* us!"—or rather *defend* us! The artificial distinctions in this country are surely minute enough, and the education sufficiently diversified—but when English sense submits to such German trammels, there will be a censor to the press—and a police officer in every lecture-room to prevent a liberal expression being pointed or spoken.

---

MEDICAL STATISTICS AND REFORM.

The leading article of the Medical Gazette, No. 325, gives a comparison of medical statistics in London, Paris, and Berlin, which strikingly exemplifies and supports the doctrine which we have long preached—viz. the propriety of raising the scale of general medical education, so that all, or nearly all, should possess medical or surgical degrees. The article in our contemporary is, indeed, the most reasonable and liberal that we have lately perused in that Journal, and, though he still has a fling at those who would "level upwards," the whole tenour of the facts and observations adduced by him, goes to sanction the mode of levelling which we have advocated.

In London, then, with a population of a million and a half, we have 225 graduate physicians and 1525 surgeons and general practitioners—total, 1750—or one medical practitioner to 857 of the population.

In Paris, the population is 935,108, and that capital supports 925 graduate physicians, 36 doctors of surgery, and 12 officiers de santé—total, 1084, or one to 862.

In Berlin, the population is only 240,086, while the physicians are 228, surgeons, 74 —total, 302, or one to 825.

Now it will not be said that the doctorate is more easy of access, or the medical education inferior, in France than in Great Britain —and yet almost the whole medical corps of Paris have degrees in medicine or surgery! This holds out encouragement at home, that a law to compel all to have degrees would be very unlikely to produce a dearth of medical practitioners. We verily believe that such a law would very little diminish the number of practitioners (since all now in existence would be exempt from its influence) while it would certainly raise the elementary knowledge of the aspirants far above its present level, and thus call forth a race of more "learned Thebans," if not better practitioners, than those now occupying the field.

We do not quite agree with our contemporary, that the cause of this great inequality of ranks, in the three capitals, depends on "undue difficulties, in the present state of things, in the way of British practitioners attaining the first class of their profession." Quite the contrary. We think it is owing to undue facilities given to those who are the "physicians general" of the community. How few will graduate at Edinburgh to starve in London, restricted to the exercise of a single branch of their profession, while they can graduate in Bridge Street, and flourish as general practitioners? Raise the medical education to a par (letting talent, industry, and science go as high above the level as they please), and all will then start in the race on an equal footing, while ability will be just as sure of rising then as now.

We have been taunted by our contemporary with the attempt at "levelling upwards." We plead guilty. We hope he will be equally candid, and acknowledge that his recommendation to give greater *facilities* to the *doctorate*, in this country, is a mode of "levelling downwards." It is for the profession to decide which is the better plan.

Our contemporary dwells much on the injurious effects of equallizing the education of medical practitioners, because such equality would destroy all differences in rank and title, and, what is worse, take away all stimulus to rising *by degrees* in professional honours. As the objection is repeatedly urged, we must again and again recur to the answer. Does the equality of education, of rank and title, in any one class of the profession, diminish the stimulus to rise above the common level of that class? Are any two of the fellows, licentiates, surgeons, or general practitioners precisely on a par, in real rank or distinction? We believe not. In the same street, we shall see two FELLOWS—one estimated by the public and the profession as a LION, the other as an ASS, yet the education and rank of both are the same; two licentiates in the same neighbourhood—one sought after by patients from morning till night, the other seeking after patients—often without being able to catch them—yet the education and rank of both are identical. In the same locality we shall see two general practitioners—one driving furiously in his carriage from square to square—from nobleman to nobleman, the other eking out a wretched revenue by selling matches, cold cream, and Morrison's pills! Yet the education and rank of both are the same. In close proximity to each other, we shall see two pure surgeons, one of whom will be entrusted with operations on royalty itself—while the other would not be trusted with the carving of a goose—yet the rank and education of both are the same! We see, then, that equality of education and of nominal rank has no power—no tendency to level real rank and distinction, with the advantages accruing thence. Let us see whether the present grades of rank afford a stimulus to rise from one grade to another? What would the general practitioner gain by quitting his rank, and becoming a licentiate? In nine cases out of ten, he would gain—*the loss of his practice!* Hence, no one ever attempts to climb such a giddy height—unless he has previously realized an independence, and only longs for the otium cum dignitate—with a new plate over his door.

What does the licentiate gain by admission to the fellowship? Not increase of real rank, reputation, or wealth, the grand objects of pursuit in this world—but, on the contrary, he often gains the disrespect, if not the contempt, of the class which he deserts and of that which he joins! In our humble opinion, the present ascending and descending series of ranks and titles, in a profession, the *exercise* of which is the same throughout, rather checks than augments the stimulus to real distinction. Institute imaginary or artificial honours as substitutes for real ones, and mischief is the result. Does real rank or distinction appertain to the diploma with which we start in the exercise of our profession, or to the honours which we afterwards acquire—and the additions which we make to the science which we profess? Yet the present distinctions and titles of the different grades have no necessary connexion with, or dependence on, the greatest talent or the most profound learning. A general practitioner may be a Hippocrates in the knowledge of diseases, and a Haller in medical science and literature; yet will he never be raised by the licentiates into their third heaven—nor by the FELLOWS into their paradise! We beg pardon—a solitary exception occurred in our own time. An apothecary became a licentiate—lived a long life as a licentiate—but *died* a fellow!!

But, as our aristocratic brethren have such an insatiable thirst for grades of rank, and for distinctions of title, in our indivisible art and mystery, we should be inclined to concede these external decorations to them, as we give toys and rattles to spoiled children. All we would ask in return would be this—that the *medical* education of the *plebeian* practitioner should be raised to the present level of the *patrician*—which, we humbly conceive, is not a Utopian project, seeing what is done in other countries—and this granted, we would willingly confer on the patrician orders of our noble profession, the most magnificent titles and distinctions that were ever engendered in oriental imaginations. Thus, the graduates of Oxford and Cambridge might be entitled the stars of the firmament, or the children of the SUN—while those of London, Edinburgh, Dublin, or Paris, might take the humbler appellations of nephews of the moon, or *satellites* of the earth, being strictly enjoined to revolve at respectful distances from the superior bodies. The pure surgeons might claim the designations of thunderbolts of heaven, scythes of time, or scissors of fate, according to their rank in the noble art of vivisection. These titles and distinctions, we submit, would be a fair equivalent for permission to drink at the same fountains of knowledge with themselves, and slake our thirsts *ad libitum*.

But in a country like this, where there is such a love of title, and such a horror of equality, *even in medical science*, we candidly confess that our expectations of thorough reform are very limited. We should certainly have no fear of any bad consequences arising from a perfect equality of education and title throughout the whole profession, knowing, as we do, that individual talent and industry would always keep up the greatest extent of real distinction in our ranks, were all artificial designations of superiority and inferiority abolished. But, as a great proportion—perhaps a majority, of our brethren will not subscribe to this system of "levelling upwards," we must accept such a system as our legislators shall think fit to give us.

Meanwhile, we would venture to throw out a brief sketch of the modifications that we think might be engrafted on the present divisions or classes of the profession.

*First*; we would suggest that no one should enter on the career of his medical studies before the age of eighteen, when, if designed for general practice, he should undergo an examination in classical and general knowledge prior to his apprenticeship. This apprenticeship we would limit to two years—especially in the country, where lectures could not be attended. At the age of twenty, his general medical studies should commence, and be pursued through three academic years in any recognized school within the limits of Great Britain, allowing one or two years of that time, if desired, to be spent in any proper foreign school. At the age of 23, a public examination in medicine, surgery, midwifery, pharmacy, &c. should be passed before a faculty or board, composed of physicians and surgeons. This rigid tentamen over, he should have a diploma or degree conferred on him, with the designation of "ORDINARY PHYSICIAN," entitling him to practise any or all branches of the profession *ubique terrarum*, or at least throughout the British dominions.

*Secondly*, those who aimed at a higher grade, and who wished to earn it by a more extended and expensive education, general and medical, might be indulged—and possibly without disadvantage. Such aspirant should not be allowed to enter on the professional curriculum till the age of 20, at which period he should undergo a strict examination in Greek, Latin, mathematics, and general knowledge. His medical curriculum should embrace six, or at the least five, years of clinical and preceptorial studies, two of which might be allowed to foreign schools. His examination should be the strictest of all, and in public, after which he might go out either in physic or surgery, as he pleased—and have a degree, conferring on him the title of "GRADUATE PHYSICIAN" in the one case, or "DOCTOR in SURGERY" in the other—or, if both, "DOCTOR in MEDICINE and SURGERY."

Thus there would still be two, if not three designations; but the education of all would experience a very considerable approach to amalgamation. The "ORDINARY PHYSICIAN should always have the option of passing into the class of PHYSICIAN EXTRAORDINARY, or GRADUATE, by addition of study, or rather by undergoing the examination imposed on the latter originally.

As men may work with their own tools, so the ordinary physician might be permitted to furnish his own medicines to his own patients, but not to sell them, and not to charge for them as a whole, but as a part of the remuneration for skill and attendance. This will be the most difficult point to settle, whether by the legislature or the individual practitioner. We are as firmly convinced as ever, that the general practitioner, or, as we propose to designate him in a new order of things, the PHYSICIAN in ORDINARY, will never attain the height of respectability and of satisfaction in his avocations, till he can charge *entirely* for his skill, without having any thing to do with the actual dispensing of medicine. But, as opinion runs contrary to this, especially in the country, let opinion have its way. A day will come, we apprehend, when the practice of physic and surgery will be entirely independent of the practice of pharmacy. Till then, some improvement may, perhaps, be made on the present plan. Suppose the legislature were to enable the general practitioner to charge from 3*s*. 6*d*. to 5*s*. for his daily attendance, or, where daily attendance is not necessary, for his separate visits—with a margin of 1*s*. 6*d*. to 2*s*. 6*d*. for medicines furnished, according to the nature of the case;—we think that such a scale of remuneration would be infinitely preferable to the present one, where the whole expense of a sickness comes in at the end of the year, in the form of a bill of parcels, throwing the medical skill and attendance entirely out of sight, and merging all in the gross, and often nauseous, *materiel* which the patient has swallowed during his confinement!! Is it possible that any liberal mind—that any man who has an idea above that of a tallow-chandler, or who wishes to see every gradation of medical society elevated in general estimation, can sanction or advocate such a system as that now in general use? For our own parts, we care not whom it may offend (for we can have no *personal* interest in the case) but we shall not cease to urge our brethren and the legislature to a revision and reformation of this crying evil. We do not speak from *theory* on this subject, but from ample experience and observation; and we hope to see the day yet when our recommendations shall come into operation. We may mention, en passant, the plan which we have mentioned above has been acted on successfully by several practitioners within the circle of our own acquaintance—and that the plan of prescribing *exclusively*, without furnishing any medicines, has also been adopted with complete success, by more than one or two general practitioners in the immediate vicinity of London. We apprehend that what can be done in a few instances, may be done in a multitude of cases. It requires, however, a simultaneous movement in the ranks.

*Thirdly*. An important question remains to be decided by the "collective wisdom." What is to become of our corporate bodies—the College of Physicians, the College of Surgeons, and the Worshipful College of Apothecaries? In metaphysics we admire the Pythagorean doctrine of transmigration, rather than that of annihilation; and in physic, we would follow something of the same doctrine. We would amalgamate the Colleges of Physicians and Surgeons, their disciples being all in the practice of one and the same art and science—and we would confine the Worshipful Company of Apothecaries to the "*statu quo*"—to the counter—to the art and mystery of pounding and compounding DRUGS, instead of pounding and compounding DOCTORS. We can see no reason why the two Colleges should not be incorporated into ONE FACULTY, furnishing a Court of Examiners for all candidates, and constituting the ruling body of the profession.

If the two divisions, to which we have alluded, should be adopted, we would concede to the GRADUATE Doctors of Physic and Surgery, the election of two-thirds of the ruling body from their own class, and one third from the ordinary physicians. This plan would give a preponderance to the higher grade, while it held out encouragement to the lower to rise—and at all events, gave them a voice in the medical council.

As all medical practitioners must necessarily belong to this FACULTY, so, a moderate fee from each would bring in a princely revenue to this same Faculty, while the honour of forming a component part of the cabinet, or ruling body, would prove a most powerful stimulus to industry and study.

The amalgamation of the Colleges in Edinburgh and Dublin would follow, of course; these would constitute parts of the same faculty, and be obliged to enforce the same curricula of education, &c. as the parent faculty in the metropolis.

With the Universities, whether English, Irish, or Scotch, there need be no interference. Those who go to Oxford or Cambridge for Greek, Latin, mathematics, and aristocratic connexions (for physic is out of the question in those seminaries) should still be allowed to reap all the advantages which universities furnish, and the degrees conferred there should be attached to their other honours—but they ought to have no exclusive privileges over their brethren. The knowledge acquired on the banks of the Thames, between Battersea and Blackfriars, is just as good, (if to the same amount) as that which is acquired on the banks of the same river, when a lazy streamlet running through Oxfordshire.

Some of our contemporaries object to this "levelling upwards" in the medical profession, on the plea that the poor in particular, and even the inhabitants of country villages in general would be left without medical assistance, since no one who incurred the expenses of an education such as we propose, would settle in villages, hamlets, or sequestered rural situations. We have no right to doubt the sincerity of these reasoners, or that they are actuated by any other motives than those of philanthropy towards society at large, and humanity towards the indigent and afflicted. But we have a right to question the legitimacy of their arguments and deductions. On this, their *point d'appui* of objection to "levelling upwards," we meet them without the least apprehension, or rather with the fullest confidence. As the experiment has not yet been tried in this country, we can only argue by analogy—or by what has been done in other countries. We need only refer to the statements made in our contemporary, to shew that in Paris, nine-tenths of the medical practitioners are physicians, with a curriculum more extended than that of physicians in this country. Where then is the impracticability of raising the standard of medical education at home, when we see it done so easily abroad? Have Englishmen less means, or less capacity for knowledge than Frenchmen? But let us draw the analogy closer to our own doors. Our contemporary is too pious to agree that the cure of our immortal souls is of less consequence than that of our perishable bodies. Yet our bishops and our legislators have not feared that the spiritual concerns of our villages and hamlets would be sacrificed by making a *University education* a preparatory step to clerical duties. We would ask whether the most retired country church ever stands empty for want of a curate on 70 or 100 pounds a year, although that curate is obliged to produce proofs of a university education? In fact, we are perfectly confident, that if every member of the medical profession were compelled to graduate at Oxford or Cambridge, previous to practice, such is the redundancy of population, and the difficulty of finding employments for the youth of this country, there would not be a village in England without a doctor. It is very probable, indeed, that there would be fewer medical practitioners than at present. There might be only ONE in a village, instead of half a dozen—but that one would be more respectable, and he would not be obliged to descend to low and disreputable means of supplanting his rivals, and pulling the bread out of their mouths to keep himself and family from starving.

We firmly believe that, if the education and title of the present GENERAL PRACTITIONER were raised to that of the physician —if quacks were put down—and chemists restrained to their shops—the whole profession would assume a more respectable character—the public health would be better preserved—and the indigent classes more generously relieved than at present.

---

### Northern Provincial Meeting of Medical Practitioners in Ireland.

The province of Ulster, the most intelligent and intellectual province of Ireland, has set a striking example to their brethren on both sides of the Irish Channel, by a general meeting of influential practitioners in Belfast, where a string of resolutions were passed, and several eloquent speeches made on the subject of Medical Reform. The corporate bodies came in for their share of castigation. We regret that we cannot afford space for more than one or two out of eleven resolutions passed at one of the largest meetings that perhaps took place on the subject of medical reform, beyond the precincts of the metropolis.

*Resolved* 1*st*. That many of the laws which now govern the Medical Profession, in these kingdoms, are unjust, partial, inefficient, and not adapted to the present condition of society, or to the advanced state of Medical Science; and several of the Medical Corporations, which have had legal powers entrusted to them, have hitherto used these as instruments of oppression and self-aggrandisement, to the great injury of the Profession, and in opposition to the public good.

*Resolved* 4*th*. That the wants of society in later years, have created a demand for, and require, the *General* Medical Practitioner, who necessarily officiates in every deparment of the healing art—as Physician, Surgeon, and Apothecary; and therefore that all distinctions of grade or name are antiquated, unnecessary, injurious to the profession, by introducing invidious grounds of fancied superiority among its members; and by varying the course of education, are detrimental to the well-being of the community at large.

*Resolved* 5*th*. That, as it appears, from several sections of the proposed "New Act" of the "Dublin Apothecaries' Company," which are evidently intended to operate retrospectively, they would oppress and impose fines on the General Practitioners, now in business, for pursuing that part of their profession called *Pharmacy,* by a galling system of espionage and gross injustice, demonstrating that their own pecuniary interests, and not the good of the medical profession, or of the public, are now, as they always have been, their sole end and aim—we will oppose their intentions by every legal means in our power.

We may take this opportunity of entreating our provincial brethren not to desert their post, or forsake the good cause of reform, because a parliamentary enquiry is instituted. The recommendations of the committee, whatever they may be, are only preliminary, and the plan of reform must undergo a fiery ordeal in both Houses of Parliament. If therefore the general sense of the medical community be not expressed universally, through the medium of petitions from the country, we stand a chance of being ultimately defeated in the practical stages of the question. Our country cousins should recollect, that we of the metropolis are not omnipotent, and that our exertions should be aided by their voice and support. They will have great reason to regret their supineness, if they leave us in the lurch on this occasion.

Sat verbum sapientibus!

Obituary.

It escaped us to notice the death of the late Sir William Franklin, of the Army Medical Board, on the 27th of October last. We regret this the less, as we understand that his son-in-law, the Rev. Mr. Bennett, is drawing up a short biographical notice of this amiable, learned, and accomplished medical officer. Of this biography we shall take due notice; and, in the mean time have to state that his friends have entered into a subscription to erect a monument to his memory. Never was memorial of merit more justly due, or more richly deserved than on this occasion.

